# Is nonalcoholic fatty liver disease associated with the development of prostate cancer? A nationwide study with 10,516,985 Korean men

**DOI:** 10.1371/journal.pone.0201308

**Published:** 2018-09-19

**Authors:** Yoon Jin Choi, Dong Ho Lee, Kyung-Do Han, Hyuk Yoon, Cheol Min Shin, Young Soo Park, Nayoung Kim

**Affiliations:** 1 Department of Internal Medicine and Seoul National University Bundang Hospital, Seongnam, Gyeonggi-do, South Korea; 2 Department of Internal Medicine, Korea University Guro Hospital, Guro-gu, Seoul, South Korea; 3 Department of Internal Medicine and Liver Research Institute, Seoul National University College of Medicine, Seoul, South Korea; 4 Department of Biostatistics, College of Medicine, The Catholic University of Korea, Seoul, South Korea; Universita degli Studi di Verona, ITALY

## Abstract

**Background:**

Growing evidence supports that prostate cancer (PCa) is a metabolic syndrome-related cancer, but the evidence is lacking regarding the association between nonalcoholic fatty liver disease (NAFLD) and PCa. We aimed to investigate whether PCa is related with NAFLD in Korean adults.

**Methods:**

Data from the National Health Insurance Corporation between 2009 and 2012 were analyzed using multivariate logistic regression method. NALFD was defined based on the fatty liver index (FLI) and hepatic steatosis index (HSI). Newly diagnosed PCa was identified using the claims data.

**Results:**

NAFLD based on FLI and HSI was identified in 2,002,375 (19%) and 2,629,858 (25%) of 10,516,985 subjects, respectively. Each FLI ≥ 60 and HSI ≥ 36 was independently associated with the development of PCa after adjusting for other confounders (hazard ratio (HR) 1.09, 95% CI: 1.06–1.12 and HR 1.19, 95% CI: 1.16–1.23). The association was more prominent among those who were older (FLI, ≥ 65 years old and HSI, ≥ 40 years old), were not currently smoking, were presently consuming alcohol (< 30g/day) and had null components of metabolic syndrome than each counterpart. Non-obese persons with NAFLD defined by HSI had a higher risk of developing PCa than those with body mass index > 25 Kg/m^2^.

**Conclusions:**

NAFLD defined by FLI or HSI may help identify high-risk individuals for developing PCa particular in the elderly, even in the absence of obesity or metabolic syndrome. Future studies on this topic should necessarily be repeated based on ultrasonographic findings.

## Introduction

Prostate cancer (PCa) is the second most commonly diagnosed cancer and the sixth most common cause of cancer-related mortality among men worldwide [1]. Although localized PCa usually has a good prognosis, prostatectomy often results in complications, such as erectile dysfunction or urinary incontinence that dramatically deteriorates the quality of life [2]. Nonetheless, the only established risk factors for PCa are age, race, and family history [3]. Regarding race, Asian men have a far lower incidence of PCa than those in Western countries, and the large geographic disparity in the incidence implies that lifestyle factors may contribute to the etiology of the disease [3].

Nonalcoholic fatty liver disease (NAFLD), which has become one of the main liver diseases worldwide [4], is closely associated with insulin resistance and metabolic syndrome. The hepatic accumulation of triacylglycerol is accompanied by abnormal hepatic metabolism and impaired insulin-mediated suppression of hepatic glucose leading to hyperglycemia, hypertriglydemia, and hyperinsulinemia [5]. Recently, the association between NAFLD and PCa has been postulated by increasing occurrences of both PCa and NAFLD, suggesting that westernization is an important risk factor for PCa [6].

There has been growing evidences that supports the hypothesis that metabolic syndrome is involved in the development and progression of certain types of malignancies [7], including PCa [8]. Given that the close association between metabolic syndrome and NAFLD, NAFLD can share common risk factors for PCa. The association of NAFLD with other extra-hepatic cancers is less proven compared with metabolic syndrome [9]. Concerning PCa, only two studies have evaluated the relationship between NAFLD and the development of PCa [10, 11].

On the grounds of this background, this study aimed to evaluate whether the NAFLD is associated with the development of PCa using nationwide data in Korea, where the incidence of both diseases is rapidly growing [12].

## Materials and methods

### Data source and study population

We used the database of the National Health Insurance Corporation (NHIC), which is a national insurer managed by the Korean government and to which approximately 97% of the Korean population subscribes [13]. The NHIC recommended subscribers to undergo a standardized medical examination at least biennially. Any researcher can use the NHIC database if the official review committee approves the study protocols.

A diagnosis of PCa was defined using the International Classification of Diseases, 10th revision (ICD-10) codes (C61) and reimbursement code for severe disease.

**Fig 1:**
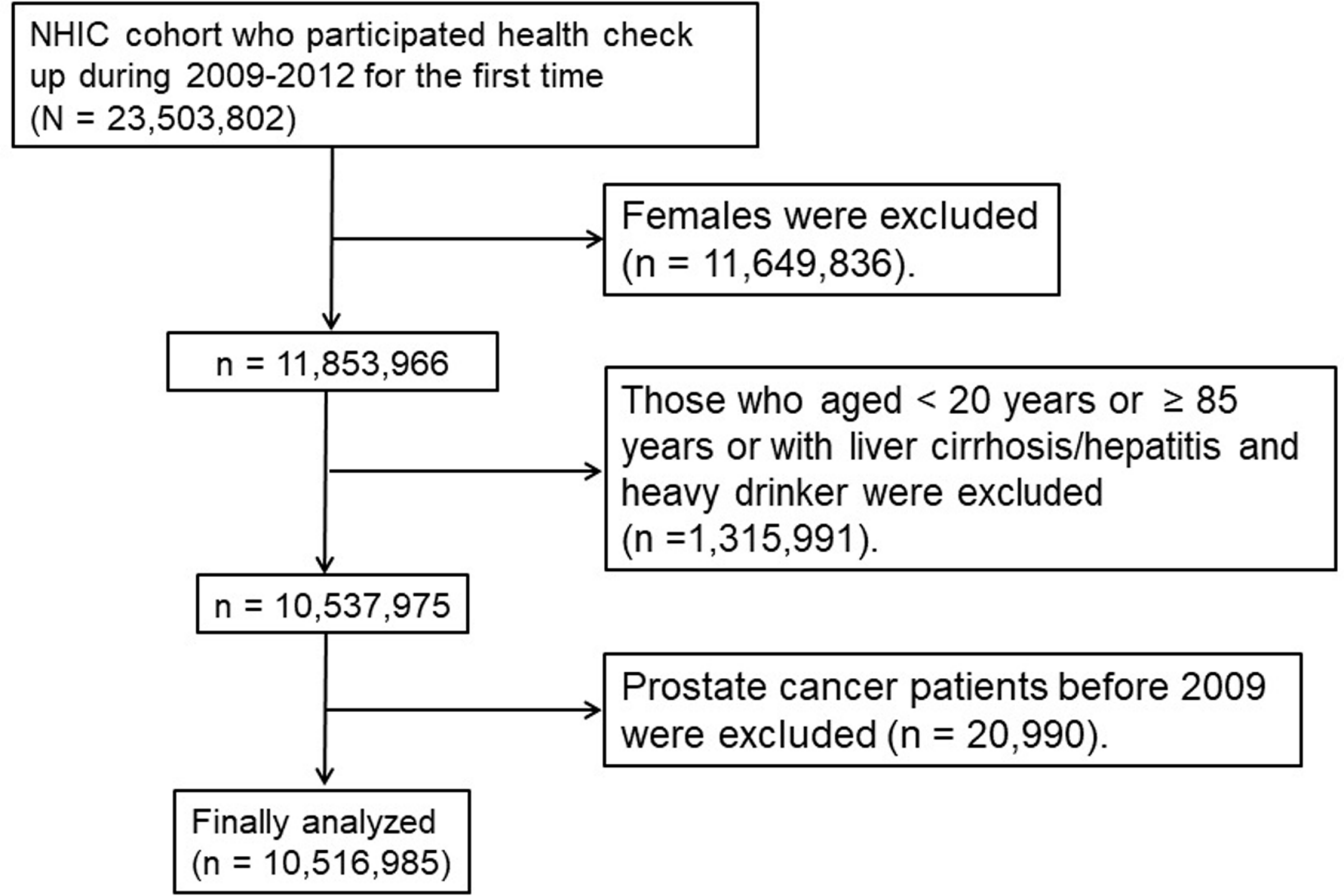
Flowchart showing the enrolment process for the study cohort. NHIC, National Health Insurance Corporation.

Among 23,503,802 individuals who had undergone an annual or biennial evaluation provided by the NHIC between the years, 2009 and 2012, 11,649,836 female subjects were excluded. Then, the study population was restricted to 11,853,966 male subjects. Those aged less than 20 years or older than 85 years and those diagnosed as having liver cirrhosis (K703) or any hepatitis (K746), and heavy alcohol consumers (≥ 30 g of alcohol per occasion in men) were excluded. After excluding 20,990 subjects who had been diagnosed as having PCa or other malignancy before 2009, 10,516,985 subjects were finally analyzed. A summary of the study population selection is illustrated in Fig 1.

### Measurements of clinical parameters and biochemical analysis

Standardized self-reporting questionnaires were used to collect data at the time of enrollment for the following variables, which are regarded as risk factors for liver injury and were included as covariates in multivariable analyses: age (years), sex, residence (rural and urban), yearly income (lower quintile vs. the remaining quintiles), alcohol intake (The frequency of alcohol consumption in 1 week and the amount of alcohol consumed on one occasion were evaluated (frequency: 0–7 days/week and amount: drinks per occasion), and cigarette smoking (never, former, and current). Regular exercise was defined as engaging in vigorous exercise on a regular basis (≥ high intensity of activity ≥ 3/week or moderate intensity of activity ≥ 5/week) [14]. Body mass index (BMI) and systolic and diastolic blood pressure (mmHg) were also measured. Subjects were considered obese when the BMI was ≥ 25 kg/m^2^ based on the criteria of the Asian-Pacific region [15]. Waist circumference ≥ 90 cm in men was defined as abdominal obesity [16]. Diabetes mellitus was defined based on using insulin or oral hypoglycemic agents, or a fasting plasma glucose level ≥ 126 mg/dL. Participants were diagnosed as being hypertensive if the systolic pressure was ≥ 140 mmHg, if the diastolic pressure was ≥ 90 mmHg, or if a current antihypertensive medication was used. After overnight fasting for at least 8 hours, blood specimens collected from each subject were processed and transported in cold storage to the Central Testing Institute (Neodin Medical Institute, Seoul, Korea). All the blood samples were analyzed within 24 hours after transportation. The serum levels of creatinine and the lipid and liver enzyme profiles were determined using a Hitachi 7600 automated chemistry analyzer (Hitachi, Tokyo, Japan) with specific indicated methods. Values of total cholesterol (mg/dL) and liver enzymes such as alanine transaminase (ALT), aspartate aminotransferase (AST), and γ-glutamyl transferase (GGT) in the serum (IU/L) were determined [17]. All procedures involving human participants were performed under the ethical standards of the institutional and national research committees, and 1964 Helsinki declaration including its later amendments or comparable ethical standards. Since the study involved routinely collected data, informed consent was not specifically obtained for this study. The study was approved by the Institutional Review Board of Seoul National University Bundang Hospital (X-1608/360-906).

#### Ethical approval

All procedures involving human participants were performed in accordance with the ethical standards of the institutional and national research committees, and the 1964 Helsinki declaration including its later amendments or comparable ethical standards. Informed consent for using information was exempted by the institutional and national research committees because this study used only previously collected data.

The funders had no role in study design, data collection and analysis, decision to publish, or preparation of the manuscript.

#### Surrogate measure of fatty liver

Although ultrasonography is a first-line screening technique and the best proxy for the invasive liver biopsy, an only small portion of enrollees underwent this in NHIC. Therefore, noninvasive tests such as the fatty liver index (FLI) [18] or hepatic steatosis index (HSI) were used [19] with limited accuracy. The FLI, ranging from 0 to 100, was calculated according to an algorithm based on triglycerides levels, BMI, GGT, and waist circumference [20]: FLI = (e ^0.953×Ln(triglyceride)+0.139×BMI+0.718×Ln(GGT)+0.053×waist circumference–15.745^)/ (1+e ^0.953×Ln(triglyceride)+0.139×BMI+0.718×Ln(GGT) +0.053×waist circumference–15.745^)×100.

In this study, subjects were then categorized into three FLI groups: < 30; 30 to 59 and ≥ 60 based on a previous studies [20, 21] Subjects were classified as having NAFLD if the FLI was ≥ 60 in the absence of other causes of chronic liver disease (e.g., a history of hepatitis or cirrhosis, hepatitis B surface antigen negative, and excessive alcohol consumption, as defined previously). As a supplementary criteria, the HSI = 8×(ALT/AST ratio) + BMI (+2, if female; +2, if diabetes mellitus) was calculated [19].

### Statistical analyses

Data are presented as the mean±standard deviation for normally distributed continuous variables and as proportions for categorical variables. The Student t test and analysis of variance were used to analyze continuous variables, and the differences between nominal variables were compared with the chi-square test. With regard to non-normally distributed variables, log transformations were performed. Among variables with a *P* value of less than 0.05 in univariate analyses, those with clinical importance were subjected to multivariate analyses. The incidence rates of cancers were calculated by dividing the number of events by person-time at risk. To determine the independent association of the FLI with the risk of cancer incidence, the Cox proportional hazards model was used after adjusting for age, smoking status, alcohol consumption, exercise, income, diabetes, hypertension and dyslipidemia. Subgroup analyses were performed according to age, smoking status, drinking habit, diabetes, metabolic syndrome components, obesity, abdominal obesity, physical activity, and yearly income.

Statistical analyses were performed using SAS version 9.4 (SAS Institute, Cary, NC, USA) and R version 3.2.3 (The R Foundation for Statistical Computing, Vienna, Austria, http://www.Rproject.org). A two-sided *p* value less than 0.05 was considered statistically significant. The cut-off of *p* for interaction was 0.15.

## Results

### Demographic characteristics

Of 10,516,985 Korean men included in the analysis, 50,284 (0.48%) developed PCa. The respective mean and median time to follow-up was 5.33 ± 1.16 years and 5.6 years (interquartile range [IQR]: 4.55–6.25). The geometric mean and median of the FLI value were 25.0 (95% CI 24.99–25.02) and 29.4 (IQR: 13.70–52.70). Those of HSI were 32.4 (95% CI 32.42–32.43) and 32.3 (IQR: 29.10–36.00). The prevalence rates of NAFLD based on the FLI and HSI were 19.0% and 25.0%, respectively. The total population was divided into two groups based on their FLI and HSI, respectively, and the baseline characteristics of the study participants are shown in (Table 1). Clinical, anthropometric, and metabolic variables were analyzed according to the FLI and HSI grouping.

**Table 1:** Demographics of the study enrollees.

Variables	Fatty liver index (FLI)			Hepatic steatosis index (HSI)		
	FLI < 60 (n = 8,514,610)	FLI ≥ 60 (n = 2,002,375)	p-value	HSI < 36 (n = 7,887,127)	HSI ≥ 36 (n = 2,629,858)	p-value
Age (year)	46.4 ± 14.5	46.5 ± 12.1	<0.001	47.1 ± 14.6	44.3 ± 12.2	<0.001
< 40	3,059,062 (35.9)	621,878(31.1)		2663100 (33.8)	1,017,840 (38.7)	
40, 65	4,332,630 (50.9)	1,208,514(60.4)		4,106,786 (52.1)	1434358 (54.5)	
≥ 65	1,122,918 (13.2)	171,983(8.6)		1,117,241 (14.2)	177,660 (6.8)	
Current smoker	3,525,822 (41.4)	970,741(48.5)	<0.001	3,327,520 (42.2)	1,169,043 (44.5)	<0.001
Alcohol consumption^a^	5,208,303 (61.2)	1,428,035(71.3)	<0.001	4,999,483 (63.4)	1,636,855 (62.2)	<0.001
Exercise^b^	1,736,044 (20.4)	352,837(17.6)	<0.001	1,625,908 (20.6)	462,973 (17.6)	<0.001
Lower quintile of yearly income	1,500,076 (17.6)	345,024(17.2)	<0.001	1,419,313 (18.0)	425,787 (16.2)	<0.001
BMI (Kg/m^2^)	23.3 ± 2.5	27.6 ± 2.8	<0.001	23.1± 2.4	27.3 ± 2.7	<0.001
BMI ≥ 25 Kg/m^2^	2,184,407 (25.7)	1,676,258 (83.7)	<0.001	1,696,006 (21.5)	2,164,659 (82.3)	<0.001
WC (cm)	81.5±6.7	92.2 ± 6.7	<0.001	81.3 ± 6.8	90.4 ± 7.0	<0.001
Abdominal obesity^c^	985418 (11.6)	1293758 (64.6)	<0.001	887,049 (11.3)	1,392,127 (52.9)	<0.001
SBP (mmHg)	123.1±13.7	129.7 ± 14.2	<0.001	123.3 ± 14.0	127.5 ± 13.8	<0.001
DBP (mmHg)	76.8±9.4	81.6 ± 9.9	<0.001	76.9 ± 9.5	80.0 ± 9.7	<0.001
Diabetes	734,276 (8.6)	357,436 (17.9)	<0.001	588,195 (7.5)	503,517(19.2)	<0.001
Hypertension	2,007,695 (23.6)	850,134 (42.5)	<0.001	1,931,622 (24.5)	926,207 (35.2)	<0.001
Dyslipidemia^d^	1,211,270 (14.2)	658,228 (32.9)	<0.001	1,162,206 (14.7)	707,292 (26.9)	<0.001
≥1 of metabolic syndrome component^e^	2,930,397 (34.4)	1243657 (62.1)	<0.001	2,761,789 (35.0)	1,412,265 (53.7)	<0.001
Glucose (mg/dL)	97.3 ± 22.6	107.2 ± 31.5	<0.001	96.9 ± 21.9	105.9±31.2	<0.001
Cholesterol (mg/dL)	189.9 ± 34.5	209.9 ± 38.7	<0.001	190.6 ± 35.1	202.8±38.0	<0.001
Triglyceride (mg/dL) ^f^	109.9 (109.9, 109.9)	233.5 (233.4, 233.7)	<0.001	115.8 (115.8, 115.9)	166.4 (166.3, 166.5)	<0.001
AST (IU/L^)^^f^	23.2 (23.2, 23.2)	39.8 (39.7, 39.8)	<0.001	21.8 (21.8, 21.8)	42.1 (42.0, 42.1)	<0.001
ALT (IU/L)^f^	24.4 (24.4, 24.4)	31.9 (31.9, 32.0)	<0.001	24.7 (24.7, 24.7)	28.7 (28.7, 28.7)	<0.001
GGT (IU/L)^f^	29.36(29.4, 29.4)	71.8 (71.8, 71.9)	<0.001	30.9 (30.8, 30.9)	50.0 (50.0, 50.0)	<0.001
Developing PCa	42,258 (0.5)	8,026 (0.4)	<0.001	41,162 (0.5)	9,122 (0.4)	<0.001
F/U duration (year)	5.4 ± 1.2	5.3 ± 1.2	< 0.001	5.3 ± 1.2	5.3 ± 1.1	NS

AST aspartate aminotransferase, ALT alanine transaminase, BMI body mass index, SBP systolic blood pressure, DBP diastolic blood pressure, FLI fatty liver index, F/U follow up, GGT gamma glutamyltransferase, HSI hepatic steatosis index, PCa prostate cancer, SD standard deviation, WC waist circumference, NS not significant.

Variables are expressed as mean ± SD or n (%).

^a^ Men who consumed alcohol ≥ 30g/day were initially excluded

^b^ Persons who did not perform high intensity of activity ≥ 3/week or moderate intensity of activity ≥ 5/week

^c^ Waist circumference ≥ 90cm for men

^d^ Triglyceride ≥ 150 mg/dL or user of lipid lowering drug

^e^ Having more than 1 of component among hypertension, dyslipidemia and diabetes mellitus

^f^ Geometric mean (95% confidence interval).

The FLI ≥ 60 group included more persons who smoked currently, consumed alcohol, and exercised less frequently. Moreover, subjects with an FLI ≥ 60 had higher blood pressure and higher values of fasting glucose, triglycerides, total cholesterol, AST, ALT, GGT, and BMI compared with those in the FLI< 60 group. Regarding HSI, HSI≥ 36 group included more persons who smoked currently, exercised less frequently and had higher blood pressure and higher values of fasting glucose, triglyceride, total cholesterol, AST, ALT, GGT and BMI compared with those in the other group.

Among 2,002,375 men with FLI ≥ 60, 8,026 (0.4%) persons were diagnosed with PCa, while 42,258 (0.5%) persons developed PCa of 8,514,610 non-NAFLD individuals. The characteristics of these two groups according to the development of PCa were listed in S1 Table.

### Multivariable analyses for risk factors associated with the development of PCa

Multivariate results of risks in developing PCa by FLI (or HSI) are shown in Table 2. After adjusting for age, the HR for the development of PCa in the NAFLD group was higher than that in the other (model 1). After controlling for age, smoking, alcohol consumption, regular physical exercise, yearly income, DM, hypertension and dyslipidemia, FLI 30–60 and ≥ 60 were associated with an increased risk for PCa (model 2 and 3). When FLI groups were divided into < 60 and ≥ 60, FLI ≥ 60 was still associated with an increased hazard ratio for PCa (model 4) (HR 1.05; 95% CI, 1.02–1.07). Regarding the HSI, a similar result was shown. That is, the higher the HSI, the higher the association with the risk for developing PCa (Table 2).

**Table 2:** Multivariable analyses of the impact of FLI and HSI on the risk of prostate cancer in the Korean population.

Group	N	Event	IR ^a^	HR (95% CI)			
				Model 1	Model 2	Model 3	Model 4
**FLI**							
< 30	5338624	25209	0.88	1 [Reference]	1 [Reference]	1 [Reference]	1 [Reference]
30–60	3175986	17049	1.00	1.13 (1.11,1.15)	1.12 (1.10,1.15)	1.10 (1.08,1.12)	
≥ 60	2002375	8026	0.76	1.14 (1.11,1.16)	1.14 (1.11,1.17)	1.09 (1.06,1.12)	1.05 (1.02, 1.07)
**HSI**							
< 30	3307795	17471	0.99	1 [Reference]	1 [Reference]	1 [Reference]	1 [Reference]
30–36	4579184	23690	0.96	1.19 (1.17,1.21)	1.17 (1.15,1.20)	1.15 (1.13,1.18)	
≥ 36	2630006	9123	0.65	1.25 (1.21,1.28)	1.23 (1.20,1.26)	1.19 (1.16,1.23)	1.09 (1.06, 1.11)

CI confidential intervals, FLI fatty liver index, HSI hepatic steatosis index, HR hazard ratios,

IR incidence rate

^a^1,000 person, year

Model 1: Adjusted for age

Model 2: Adjusted for age, smoking, drinking, regular exercise, income

Model 3: Adjusted for age, smoking, drinking, regular exercise, income, diabetes, hypertension, and dyslipidemia

Model 4: FLI and HSI groups were divided into < 60 and ≥ 60 or < 36 and ≥36, respectively.

Adjusted for age, smoking, drinking, regular exercise, income, diabetes, hypertension, and dyslipidemia

#### Effects of individual components of the FLI and HSI on the development of PCa

Table 3 shows the incidence rates and hazard ratios of PCa according to the BMI range, abdominal obesity, triglycerides level, and GGT and AST/ALT quartile that account for FLI or HSI. A BMI ≥ 23.0 kg/m^2^ increased the risk of PCa compared to a normal range of BMI, and there was a dose-dependent increase in the risk of PCa when the BMI increased (P _trend_ < 0.001). In contrast, underweight (BMI < 18.5 kg/m^2^) showed a reduced risk of PCa (HR 0.77; 95% CI 0.72–0.81) after adjusting for age, smoking, alcohol consumption, exercise, and yearly income.

**Table 3:** The impact of individual components of FLI and HSI scores on the risk for prostate cancer by multivariable analyses.

Variables	Number	Outcome	Duration	IR	HR (95% CI)^a^	P trend
BMI						< 0.001
< 18.5 Kg/m^2^	252248	1249	1300796	0.96	0.77 (0.72, 0.81)	
18.5, 23.0 Kg/m^2^	3580475	16757	19134023	0.88	1 [Reference]	
23.0, 25.0 Kg/m^2^	2823597	14659	15165239	0.97	1.16 (1.14, 1.19)	
25.0, 30.0 Kg/m^2^	3472109	16474	18521411	0.89	1.20 (1.17, 1.23)	
≥ 30.0 Kg/m^2^	388556	1145	2024372	0.57	1.26 (1.18, 1.33)	
Abdominal obesity^b^					< 0.001	
No	8237809	35575	44118188	0.81	1 [Reference]	
Yes	2279176	14709	12027653	1.22	1.15 (1.13, 1.17)	
Dyslipidemia^c^						< 0.001
No	6181691	28702	32986710	0.87	1 [Reference]	
Yes	4212159	21142	22438762	0.94	1.07 (1.06, 1.09)	
Diabetes						< 0.001
No	9425273	40424	50502501	0.80	1 [Reference]	
yes	1091712	9860	5643340	1.75	1.05 (1.03, 1.07)	
GGT^c^						< 0.001
< 21 IU/L	2600002	13157	13932884	0.94	1 [Reference]	
< 31 IU/L	2712295	14088	14590886	0.97	1.08 (1.05, 1.11)	
< 50 IU/L	2572446	12709	13779962	0.92	1.13 (1.10, 1.16)	
≥ 50 IU/L	2632242	10330	13842109	0.75	1.08 (1.05, 1.11)	
ALT/AST^c^						< 0.001
< 0.8	2643673	16950	13946366	1.22	1 [Reference]	
0.8–1.0	2463015	13489	13163352	1.02	1.10 (1.08, 1.13)	
< 1.3	2781073	12479	14906255	0.84	1.16 (1.13, 1.19)	
≥ 1.3	2629224	7366	14129868	0.52	1.14 (1.11, 1.17)	
ALT^d^						< 0.001
< 18 IU/L	2812374	15345	14900042	1.03	1 [Reference]	
< 24 IU/L	2535905	13787	13597829	1.01	1.08 (1.05, 1.10)	
< 34 IU/L	2552043	12282	13688390	0.90	1.11 (1.08, 1.14)	
≥ 34 IU/L	2616663	8870	13959580	0.64	1.10 (1.07, 1.13)	
AST^d^						0.236
< 20 IU/L	2526159	10557	13463044	0.78	1 [Reference]	
< 24 IU/L	2651440	12648	14213703	0.89	1.00 (0.97, 1.02)	
< 30 IU/L	2776167	14411	14891113	0.97	0.99 (0.96, 1.01)	
≥ 30 IU/L	2563219	12668	13577982	0.93	0.99 (0.96, 1.01)	
FLI^d^						< 0.001
< 13.7	2629246	10733	14039989	0.76	1 [Reference]	
< 29.4	2629246	14011	14123644	0.99	1.16 (1.13, 1.19)	
< 52.7	2629247	14370	14087052	1.02	1.22 (1.19, 1.25)	
≥ 52.7	2629246	11170	13895157	0.80	1.24 (1.21, 1.27)	
HSI^d^						< 0.001
< 29.1	2629307	13744	13942057	0.99	1 [Reference]	
29.1–32.2	2629183	14142	14091688	1.00	1.18 (1.15, 1.21)	
32.3–36.0	2629265	13279	14086812	0.94	1.24 (1.21, 1.27)	
≥ 36.0	2629230	9119	14025284	0.65	1.27 (1.24, 1.31)	

AST aspartate aminotransferase, ALT alanine transaminase, BMI body mass index, GGT gamma glutamyltransferase, FLI fatty liver index, HSI hepatic steatosis index, IR incidence rate, WC waist circumference.

^a^ Adjusted by age, smoking, drinking, exercise and income

^b^ Waist circumference ≥ 90cm for men

^c^ Triglyceride ≥ 150 mg/dL or user of lipid-lowering drug

^d^ The value was divided into quartile

Abdominal obesity, high serum triglycerides levels, DM, GGT level ≥21 IU/mL, ALT level ≥18 IU/ml, and ALT/AST level ≥ 0.8 were associated with an increased risk of PCa, while the serum AST level was not. Finally, an FLI ≥13.7 and HSI ≥29.1 were associated with an increased risk of PCa, and dose-dependent increments were shown.

#### Subgroup analyses according to age, smoking status, alcohol consumption, and metabolic health status

**Fig 2:**
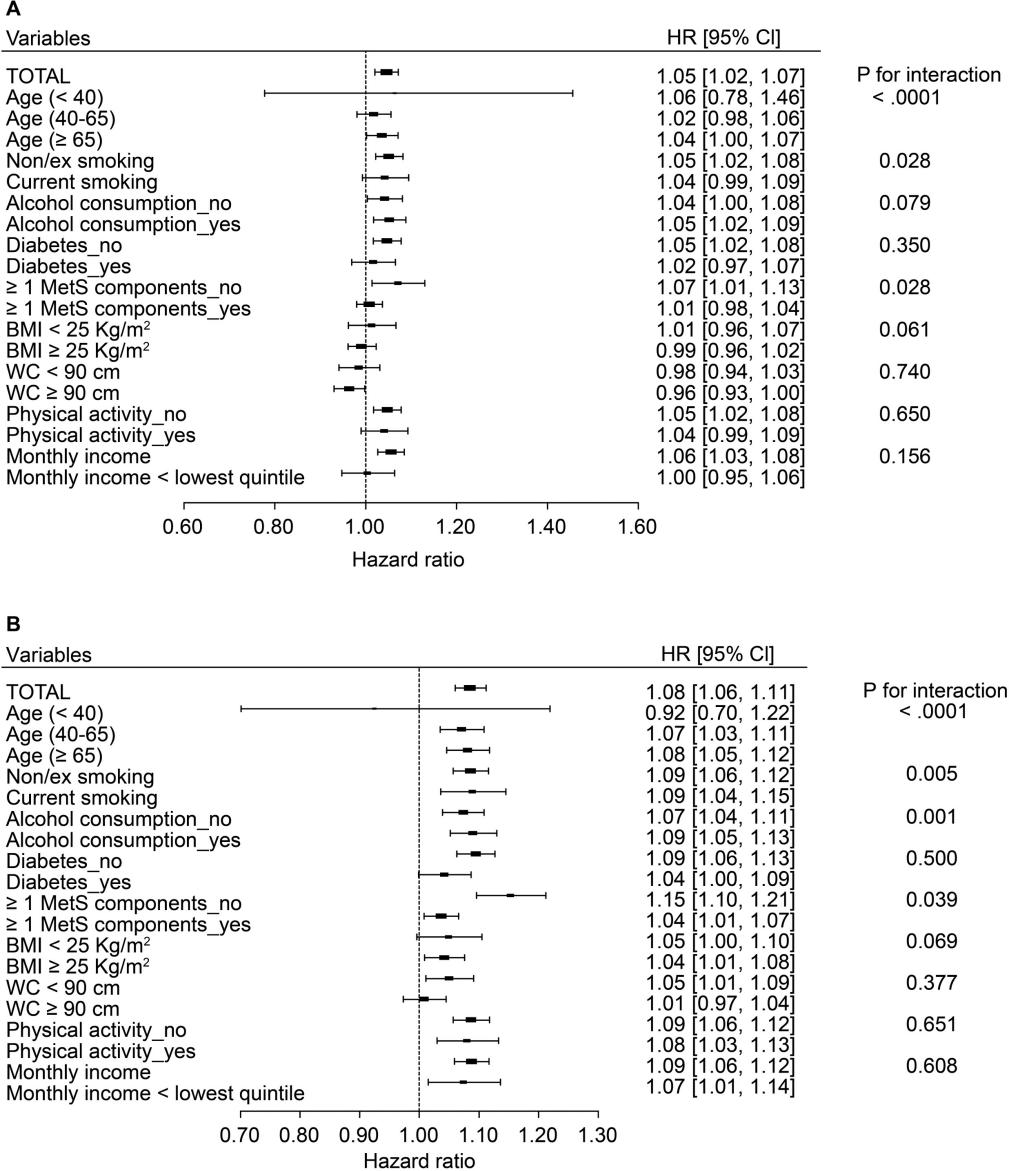
The impact of on the risk nonalcoholic fatty liver disease of prostate cancer in different subgroups. Forrest plots of hazard ratios (HR) and 95% confidential interval (CI) adjusted for age, smoking, drinking, regular exercise, income according to subgroups were illustrated. Fatty liver index (a) or hepatic steatosis index (b) was used to define the nonalcoholic fatty liver disease.

We performed subgroup analyses according to age, smoking status, alcohol consumption, DM, obesity, and the combination of hypertension and dyslipidemia. Fig 2 shows the HRs of PCa and P for interaction in each subgroup after controlling for age, smoking status, alcohol consumption, physical exercise, and yearly income.

In terms of the FLI, subjects aged 65 years or older showed the highest risk for PCa compared to other age groups (HR 1.04; 95% CI 1.00–1.07, P _interaction_ < 0.001) (Fig 2A). Current alcohol consumers (< 30 g/day for men) or non/ex-smokers and were more closely associated with developing PCa. The associations between the FLI and developing PCa were more prominent in normotensive, nondyslipidemic and normoglycemic persons than those with ≥ 1 of above three disorders (HR 1.07; 95% CI 1.01–1.13 vs. HR 1.01; 95% CI 0.98–1.04, P _interaction_ = 0.028). Each DM, obesity and abdominal obesity had a similar trend, but they did not show significant differences.

Regarding the interaction between the HSI and developing PCa, the HSI had similar results as the FLI (Fig 2B). That is, persons aged ≥ 40 years old, those who were not currently smoking or smoked in the past, those who currently consumed alcohol (< 30 g/day), and those without hypertension, dyslipidemia, and diabetes had a closer association of developing PCa than each counterpart. Non-obese persons with NAFLD defined by HSI had a higher risk of developing PCa than obese persons with NAFLD defined by HSI (HR 1.05; 95% CI 1.00–1.10 vs. HR 1.04; 95% CI 1.01–1.08, P _interaction_ = 0.069).

## Discussion

In the present study, we found that NAFLD defined by the FLI or HSI was associated with the development of PCa after controlling for age and other confounding factors. The risk of developing PCa is consistently higher with an FLI ≥ 13.7, and it steadily increased as the HSI increased ≥ 29.1. Moreover, the positive association was more prominent in persons who were older, non-current smoking, non-obese, consumed alcohol or did have none of the dyslipidemia, hypertension, or diabetes, which account for metabolic syndrome.

According to Korean cohort studies, the mean age at diagnosis of NAFLD and PCa was 41.6 and 68.7 years old, respectively [12, 22]. This discrepancy in affected ages can lead to confounding. The incidence rate of PCa is the highest in 50–60’s where that of NAFLD is decreasing. As a result, the incidence rates of PCa in NAFLD patients (by FLI > 60 or HSI > 36) during this range of age were lower than those in the non-NAFLD. However, after controlling the age, the hazard ratio of PCa in the NAFLD group is higher than that in the non-NAFLD group (Table 2). These results remain consistent after controlling several covariates besides age.

To date, only two studies have specifically investigated the association between NAFLD and PCa. In the study that compared the development of malignancies in subjects with NAFLD diagnosed by US and matched hepatitis C virus-infected patients, PCa developed in 12.6% and 3.5% in each group, respectively [10]. The other study reported that NAFLD was a protective factor against recurrence after radical prostatectomy because the NAFLD group showed a significantly longer time-to-recurrence than patients without NAFLD [11]. Colorectal cancer and pancreatic, breast, and renal cancer have been consistently reported as being associated with NAFLD [7, 23, 24]. Given that PCa has been regarded as a metabolic syndrome-associated cancer along with colorectal, pancreatic, and breast cancer [7], there is a high possibility that PCa may be associated with NAFLD, too.

The mechanisms underlying the association between NAFLD and the excess risk of PCa are not fully elucidated. The most alleged mechanism linking NAFLD and PCa is the alteration of insulin and insulin-like growth factor-I (IGF-1) similar to other cnacers [25]. It has been reported that elevated serum levels of IGF-1 were associated with PCa [26, 27].

Gender-specific manifestations of NAFLD have been consistently reported [28]. Most studies found NAFLD to be more prevalent in men [29, 30] and some studies identified different risk factors for the development of NAFLD by gender [31]. Moreover, the reproductive status also critically affects the risk of development and progression of this disease [32]. This sexual dimorphism supports a close link between endocrine derangements and the pathogenesis of NAFLD. For example, testosterone deficiency accompanied by an increased accumulation of visceral adipose tissue and insulin resistance has been observed. The lower levels of total testosterone were associated with the development of NAFLD [33, 34] and PCa [35, 36]. Besides, metabolic syndrome and NAFLD aggravated the severity of prostatic inflammation which could promote uncontrolled proliferation [37]. This hormonal basis underlying NAFLD may be the reason for the observed more prominent association with older men whose testosterone is physiologically reducing.

Interestingly, the positive associations between the NAFLD defined by HSI and developing PCa were more prominent in non-obese persons than in obese persons. The HR for PCa in NAFLD defined by FLI or HSI was also higher in the normotensive, non-dyslipidemic, and non-diabetic persons than that in individuals presenting any of them. Considering the higher prevalence of obesity or metabolic syndrome in NAFLD patients compared with healthy individuals, the stronger association between the NAFLD and the development of PCa in non-obese or persons with null-metabolic syndrome components is an unexpected result. Similar to this, a stronger association of NAFLD with the metabolic syndrome in lean individuals than in obese individuals has been previously observed [38, 39]. This might come from the diluting the powerful impact of obesity or DM on the development of PCa by dividing the population by the presence or absence of DM or obesity. This also partially reflects that BMI fails to assess the visceral fat distribution [40]. A different mechanism may underlie developing PCa in a different population, and this needs further studies.

Nonetheless, it is of note that NAFLD particularly based on HSI, could predict PCa even in the normal range of BMI. While most persons with NAFLD have insulin resistance, only a minority of them exhibit the full-blown metabolic syndrome, suggesting that NAFLD may precede the obesity or DM [38]. Indeed, it has been reported that 10% to 30% of non-obese individuals have evidence of NAFLD [39]. NAFLD may independently predict insulin resistance, identifying individuals who can be missed by criteria of metabolic syndrome [38].

The strength of our study is that it was a nationwide population-based study. While obesity has been inconsistently linked to the risk of PCa, so far [41], this study showed that FLI and HSI as well as each of obesity, dyslipidemia, abdominal obesity and DM had a positive association with an increased risk of PCa (Table 3).

One of the significant limitations of this study is that imaging modality was not used for diagnosis of NAFLD.

FLI is a proxy for ultrasonography and does not accurately quantify steatosis [42]. Semi-quantitative indices using US could diagnose with NAFLD by providing additional information on metabolic derangements and histological changes [43–45]. Other limitations include the possible misclassification of individuals who may have viral hepatitis B or C or asymptomatic compensated cirrhosis into the NAFLD group. The family history of PCa did not adjust multivariate analyses due to a lack of a family history for the specific cancer. Nevertheless, the increasing prevalence of metabolic syndrome worldwide and the high incidence of some malignancies, including PCa, support the possible association between NAFLD and the development of some cancers. Additional studies are warranted to ascertain the risk of PCa in NAFLD using semi-quantitative ultrasonographic indices and exclusion of competing causes of liver disease.

## Supporting information

S1 Table Demographics of the prostate cancer group and non- prostate cancer group. (DOCX)

## References

[1] Center MM, Jemal A, Lortet-Tieulent J, Ward E, Ferlay J, Brawley O, et al International variation in prostate cancer incidence and mortality rates. Euro Urol. 2012;61:1079–1092. 10.1016/j.eururo.2012.02.054 22424666

[2] Resnick MJ, Koyama T, Fan K-H, Albertsen PC, Goodman M, Hamilton AS, et al Long-term functional outcomes after treatment for localized prostate cancer. New Eng J Med. 2013;368:436–445. 10.1056/NEJMoa1209978 23363497 PMC3742365

[3] Gann PH. Risk factors for prostate cancer. Rev Urol 2002;4:S3. PMC1476014 16986064

[4] Charlton MR, Burns JM, Pedersen RA, Watt KD, Heimbach JK, Dierkhising RA. Frequency and outcomes of liver transplantation for nonalcoholic steatohepatitis in the United States. Gastroenterology 2011;141:1249–1253. 10.1053/j.gastro.2011.06.061 21726509

[5] Liver EAftSot, Diabetes EAftSo. EASL-EASD-EASO Clinical Practice Guidelines for the management of non-alcoholic fatty liver disease. Obesity facts 2016:9:65–90. 10.1159/000443344 27055256 PMC5644799

[6] Hsing AW, Devesa SS. Trends and patterns of prostate cancer: what do they suggest? Epidemiol Rev 2001;23:3–13. 11588851 10.1093/oxfordjournals.epirev.a000792

[7] Esposito K, Chiodini P, Colao A, Lenzi A, Giugliano D. Metabolic syndrome and risk of cancer. Diabetes care 2012;35:2402–2411. 10.2337/dc12-0336 23093685 PMC3476894

[8] Gacci M, Russo G, De Nunzio C, Sebastianelli A, Salvi M, Vignozzi L, et al Meta-analysis of metabolic syndrome and prostate cancer. Prostate cancer and prostatic diseases 2017;20:146 10.1038/pcan.2017.1 28220805

[9] Sanna C, Rosso C, Marietti M, Bugianesi E. Nonalcoholic fatty liver disease and extra-hepatic cancers. Int J Mol Sci 2016;17:717. 10.3390/ijms17050717 PMC4881539 27187365

[10] Arase Y, Kobayashi M, Suzuki F, Suzuki Y, Kawamura Y, Akuta N, et al Difference in malignancies of chronic liver disease due to non-alcoholic fatty liver disease or hepatitis C in Japanese elderly patients. Hepatol Res 2012;42:264–272. 10.1111/j.1872-034X.2011.00915.x 22175908

[11] Choi W-M, Lee J-H, Yoon J-H, Kwak C, Lee YJ, Cho YY, et al Nonalcoholic fatty liver disease is a negative risk factor for prostate cancer recurrence. Endocr Relat Cancer 2014;21:343–353. 10.1530/ERC-14-0036 24481324

[12] Heo JE AH, Kim J, Chung BH, Lee KS. Changes in Clinical Characteristics of Patients with an Initial Diagnosis of Prostate Cancer in Korea: 10-Year Trends Reported by a Tertiary Center. J Kor Med Sci 2018;33:e42. 10.3346/jkms.2018.33.e42 PMC5777916 29349937

[13] Lee J, Lee JS, Park S-H, Shin SA, Kim K. Cohort profile: The national health insurance service–national sample cohort (NHIS-NSC), South Korea. Int J Epidemiol 2016;46:e15–e15. 10.1093/ije/dyv319 26822938

[14] Lee Y-h, Kim JE, Roh YH, Choi HR, Rhee Y, Kang DR, et al The combination of vitamin D deficiency and mild to moderate chronic kidney disease is associated with low bone mineral density and deteriorated femoral microarchitecture: results from the KNHANES 2008–2011. J Clin Endocrinol Metab. 2014;99:3879–3888. 10.1210/jc.2013-3764 24878040

[15] Oh SW. Obesity and metabolic syndrome in Korea. Diabetes Metab J. 2011;35:561–566. 10.4093/dmj.2011.35.6.561 22247896 PMC3253964

[16] Lee SY, Park HS, Kim DJ, Han JH, Kim SM, Cho GJ, et al Appropriate waist circumference cutoff points for central obesity in Korean adults. Diabetes Res Clin Pract. 2007;75:72–80. 10.1016/j.diabres.2006.04.013 16735075

[17] Janssen I, Heymsfield SB, Ross R. Low relative skeletal muscle mass (sarcopenia) in older persons is associated with functional impairment and physical disability. J Am Geriat Soc. 2002;50:889–896. 12028177 10.1046/j.1532-5415.2002.50216.x

[18] Bedogni G, Bellentani S, Miglioli L, Masutti F, Passalacqua M, Castiglione A, et al The Fatty Liver Index: a simple and accurate predictor of hepatic steatosis in the general population. BMC gastroenterol. 2006;6:33 10.1186/1471-230X-6-33 17081293 PMC1636651

[19] Lee J-H, Kim D, Kim HJ, Lee C-H, Yang JI, Kim W, et al Hepatic steatosis index: a simple screening tool reflecting nonalcoholic fatty liver disease. Dig Liv Dis. 2010;42:503–508. 10.1016/j.dld.2009.08.002 19766548

[20] Lee Y-h, Kim SU, Song K, Park JY, Kim DY, Ahn SH, et al Sarcopenia is associated with significant liver fibrosis independently of obesity and insulin resistance in nonalcoholic fatty liver disease: Nationwide surveys (KNHANES 2008‐2011). Hepatology 2016. 10.1002/hep.28376 26638128

[21] Gastaldelli A, Kozakova M, Højlund K, Flyvbjerg A, Favuzzi A, Mitrakou A, et al Fatty liver is associated with insulin resistance, risk of coronary heart disease, and early atherosclerosis in a large European population Hepatology 2009;49:1537–1544. 10.1002/hep.22845 19291789

[22] Jeong EH, Jun DW, Cho YK, Choe YG, Ryu S, Lee SM, et al Regional prevalence of non-alcoholic fatty liver disease in Seoul and Gyeonggi-do, Korea. Clin Mol Hepatol 2013;19:266 10.3350/cmh.2013.19.3.266 24133664 PMC3796676

[23] Van Gaal LF, Mertens IL, Christophe E. Mechanisms linking obesity with cardiovascular disease. Nature 2006;444:875–880. 10.1038/nature05487 17167476

[24] Tilg H, Diehl AM. NAFLD and extrahepatic cancers: have a look at the colon. Gut 2011;60:745–746. 10.1136/gut.2011.239392 21454382 PMC3638235

[25] Tilg H, Moschen AR. Mechanisms behind the link between obesity and gastrointestinal cancers. Best Prac Res Clin Gastroenterol 2014;28:599–610. 10.1016/j.bpg.2014.07.006 25194178

[26] Grimberg A, Cohen P. Role of insulin-like growth factors and their binding proteins in growth control and carcinogenesis. J Cell Physiol 2000;183:1–9. 10.1002/(SICI)1097-4652(200004)183:1<1::AID-JCP1>3.0.CO;2-J 10699960 PMC4144680

[27] Chan JM, Stampfer MJ, Giovannucci E, Gann PH, Ma J, Wilkinson P, et al Plasma insulin-like growth factor-I and prostate cancer risk: a prospective study. Science 1998;279:563–566. 9438850 10.1126/science.279.5350.563

[28] Ballestri S, Nascimbeni F, Baldelli E, Marrazzo A, Romagnoli D, Lonardo A. NAFLD as a Sexual Dimorphic Disease: Role of Gender and Reproductive Status in the Development and Progression of Nonalcoholic Fatty Liver Disease and Inherent Cardiovascular Risk. Adv Ther. 2017;34:1291–1326. 10.1007/s12325-017-0556-1 28526997 PMC5487879

[29] Lonardo A, Lombardini S, Scaglioni F, Carulli L, Ricchi M, Ganazzi D, et al Hepatic steatosis and insulin resistance: does etiology make a difference? J Hepatol. 2006;44:1196–1207. 10.1016/j.jhep.2006.03.005 16168516

[30] Bertolotti M, Lonardo A, Mussi C. Baldelli E, Pellegrini E, Ballestri S, et al Nonalcoholic fatty liver disease and aging: epidemiology to management. World J Gastroenterol 2014;20:14185–14204. 10.3748/wjg.v20.i39.14185 25339806 PMC4202348

[31] Lonardo A, Trande P. Are there any sex differences in fatty liver? A study of glucose metabolism and body fat distribution. J Gastroenterol Hepatol. 2000;15:775–782. 10937684 10.1046/j.1440-1746.2000.02226.x

[32] Kim D, Kim WR. Nonobese fatty liver disease. Clin Gastroenterol Hepatol 2017;15:474–485. 10.1016/j.cgh.2016.08.028 27581063

[33] Nikolaenko L, Jia Y, Wang C, Diaz-Arjonilla M, Yee J, French S, et al Testosterone replacement ameliorates nonalcoholic fatty liver disease in castrated male rats. Endocrinology 2014;155:417–428. 10.1210/en.2013-1648 24280056 PMC5393315

[34] Mody A, White D, Kanwal F, Garcia JM. Relevance of low testosterone to non-alcoholic fatty liver disease. Cardiovasc Endocrinol 2015;4:83 10.1097/XCE.0000000000000057 26405614 PMC4577238

[35] Han JH, Choi NY, Bang SH, Kwon OJ, Jin YW, Myung SC, et al Relationship between serum prostate-specific antigen levels and components of metabolic syndrome in healthy men. Urology 2008;72:749–754. 10.1016/j.urology.2008.01.084 18701153

[36] Zhang PL, Rosen S, Veeramachaneni R, Kao J, DeWolf WC, Bubley G. Association between prostate cancer and serum testosterone levels. Prostate 2002;53:179–182. 10.1002/pros.10140 12386917

[37] Russo GI, Cimino S, Castelli T, Favilla V, Gacci M, Carini M, et al Benign Prostatic Hyperplasia, Metabolic Syndrome and Non‐Alcoholic Fatty Liver Disease: Is Metaflammation the Link? Prostate 2016;76:1528–1535. 10.1002/pros.23237 27458062

[38] Lonardo A, Ballestri S, Marchesini G, Angulo P, Loria P. Nonalcoholic fatty liver disease: a precursor of the metabolic syndrome. Dig Liv Dis 2015;47:181–190. 10.1016/j.dld.2014.09.020 25739820

[39] Kwon Y-M, Oh S-W, Hwang S-s, Lee C, Kwon H, Chung GE. Association of nonalcoholic fatty liver disease with components of metabolic syndrome according to body mass index in Korean adults. Am J Gastroenterol. 2012;107:1852–1858. 10.1038/ajg.2012.314 23032980

[40] Cusi K. Role of obesity and lipotoxicity in the development of nonalcoholic steatohepatitis: pathophysiology and clinical implications. Gastroenterology 2012;142:711–725. e716. 10.1053/j.gastro.2012.02.003 22326434

[41] McGrowder DA, Jackson LA, Crawford TV. Prostate cancer and metabolic syndrome: is there a link? Asian Pacific J Cancer Prev 2012;13:1–13. 10.7314/apjcp.2012.13.1.001 22502649

[42] Fedchuk L, Nascimbeni F, Pais R, Charlotte F, Housset C, Ratziu V. Performance and limitations of steatosis biomarkers in patients with nonalcoholic fatty liver disease. Aliment pharmacol Therapeut 2014;40:1209–1222. 10.1111/apt.12963 25267215

[43] Ballestri S, Lonardo A, Romagnoli D, Carulli L, Losi L, Day CP, et al Ultrasonographic fatty liver indicator, a novel score which rules out NASH and is correlated with metabolic parameters in NAFLD. Liver International 2012;32:1242–1252. 10.1111/j.1478-3231.2012.02804.x 22520641

[44] Yang KC, Hung H-F, Lu C-W, Chang H-H, Lee L-T, Huang K-C. Association of non-alcoholic fatty liver disease with metabolic syndrome independently of central obesity and insulin resistance. Scientific reports 2016;6:27034 10.1038/srep27034 27246655 PMC4887873

[45] Ballestri S, Nascimbeni F, Baldelli E, Marrazzo A, Romagnoli D, Targher G, et al Ultrasonographic fatty liver indicator detects mild steatosis and correlates with metabolic/histological parameters in various liver diseases. Metab Clin Experiment 2017;72:57–65. 10.1016/j.metabol.2017.04.003 28641784

