# Supplementary material for: Is nonalcoholic fatty liver disease associated with the development of prostate cancer? A nationwide study with 10,516,985 Korean men
Source: PLoS One. 2018 Sep 19;13(9):e0201308. doi: 10.1371/journal.pone.0201308 (PMC6145525; doi:10.1371/journal.pone.0201308)
Supplement: S1 Table — (DOCX) [file pone.0201308.s001.docx]

Table S1 Demographics of the prostate cancer group and non- prostate cancer group

|  | Non-PCa | PCa | p |
| --- | --- | --- | --- |
|  | 10466701 | 50284 |  |
| FLI |  |  | <.0001 |
| FLI < 60 | 8472352(80.95) | 42258(84.04) |  |
| FLI ≥ 60 | 1994349(19.05) | 8026(15.96) |  |
| HSI |  |  | <.0001 |
| HSI < 36 | 7845965(74.96) | 41162(81.86) |  |
| HSI ≥ 36 | 2620736(25.04) | 9122(18.14) |  |
| Age (years) | 46.31±14.06 | 66.16±8.62 | <.0001 |
| Age group |  |  | <.0001 |
| 20-39 | 3680681(35.17) | 259(0.52) |  |
| 40-64 | 5521655(52.75) | 19489(38.76) |  |
| 65- | 1264365(12.08) | 30536(60.73) |  |
| Current smoker | 4484319(42.84) | 12244(24.35) | <.0001 |
| Alcohol consumption ^a^ | 6612889(63.18) | 23449(46.63) | <.0001 |
| Regular physical activity ^b^ | 2075682(19.83) | 13199(26.25) | <.0001 |
| Lower quintile of  yearly income | 1835848(17.54) | 9252(18.4) | <.0001 |
| BMI (Kg/m^2^) | 24.13±3.07 | 24±2.85 | <.0001 |
| Obesity | 3843046(36.72) | 17619(35.04) | <.0001 |
| Abdominal obesity ^c^ | 2264467(21.63) | 14709(29.25) | <.0001 |
| Diabetes | 1081852(10.34) | 9860(19.61) | <.0001 |
| Hypertension | 2830919(27.05) | 26910(53.52) | <.0001 |
| Dyslipidemia ^d^ | 1856201(17.73) | 13297(26.44) | <.0001 |
| ≥1 of metabolic  syndrome ^e^ | 4141043(39.56) | 33011(65.65) | <.0001 |
| WC (cm) | 83.53±7.92 | 85.37±7.81 | <.0001 |
| Glucose (mg/dL) | 99.13±24.83 | 103.82±25.88 | <.0001 |
| SBP (mmHg) | 124.34±14.04 | 128.25±15.17 | <.0001 |
| DBP (mmHg) | 77.69±9.66 | 78.19±9.77 | <.0001 |
| Cholesterol (mg/dL) | 193.69±36.22 | 190.55±36.34 | <.0001 |
| ALT ^f^ | 25.72(25.71-25.73) | 23.53(23.44-23.62) | <.0001 |
| AST ^f^ | 25.68(25.67-25.68) | 26.01(25.94-26.08) | <.0001 |
| r-GTP ^f^ | 34.82(34.81-34.84) | 32.86(32.68-33.05) | <.0001 |
| Triglyceride ^f^ | 126.85(126.8-126.89) | 122.22(121.67-122.78) | <.0001 |
| Duration | 5.35±1.15 | 3.04±1.75 | <.0001 |

AST aspartate aminotransferase, ALT alanine transaminase, BMI body mass index, SBP systolic blood pressure, DBP diastolic blood pressure, FLI fatty liver index, F/U follow up, r-GTP gamma glutamyltransferase, HSI hepatic steatosis index, PCa prostate cancer, SD standard deviation, WC waist circumference, NS not significant.

Variables are expressed as mean ± SD or n (%).

^a^ Men who consumed alcohol ≥30g/day were initially excluded

^b^ Persons who did not perform high intensity of activity ≥ 3/week or moderate intensity of activity ≥ 5/week

^c^ Waist circumference ≥90cm for men

^d^ Triglyceride ≥ 150 mg/dL or user of lipid lowering drug

^e^ Having more than 1 of component among hypertension, dyslipidemia and diabetes mellitus

^f^ Geometric mean (95% confidence interval).
